# A Clinical Nomogram Based on the Triglyceride-Glucose Index to Predict Contrast-Induced Acute Kidney Injury after Percutaneous Intervention in Patients with Acute Coronary Syndrome with Diabetes Mellitus

**DOI:** 10.1155/2022/5443880

**Published:** 2022-10-27

**Authors:** Yue Hu, Xiaotong Wang, Shengjue Xiao, Na Sun, Chunyan Huan, Huimin Wu, Minjia Guo, Tao Xu, Defeng Pan

**Affiliations:** ^1^Department of General Practice, The Affiliated Hospital of Xuzhou Medical University, Xuzhou, Jiangsu, China 221004; ^2^Department of Cardiology, The Affiliated Hospital of Xuzhou Medical University, Xuzhou, Jiangsu, China 221004; ^3^Department of Cardiology, Zhongda Hospital, School of Medicine, Southeast University 87 Dingjiaqiao, Nanjing, Jiangsu, China 210009

## Abstract

The aim of the study was to investigate the factors influencing contrast-induced acute kidney injury (CI-AKI) after percutaneous intervention (PCI) in patients with acute coronary syndrome (ACS) with diabetes mellitus (DM). A total of 1073 patients with ACS combined with DM who underwent PCI at the Affiliated Hospital of Xuzhou Medical University were included in this study. We divided the patients into the CI-AKI and non-CI-AKI groups according to whether CI-AKI occurred or not. The patients were then randomly assigned to the training and validation sets at a proportion of 7 : 3. Based on the results of the LASSO regression and multivariate analyses, we determined that the subtypes of ACS, age, multivessel coronary artery disease, hyperuricemia, low-density lipoprotein cholesterol, triglyceride-glucose index, and estimated glomerular filtration rate were independent predictors on CI-AKI after PCI in patients with ACS combined with DM. Using the above indicators to develop the nomogram, the AUC-ROC of the training and validation sets were calculated to be 0.811 (95% confidence interval (CI): 0.766-0.844) and 0.773 (95% CI: 0.712-0.829), respectively, indicating high prediction efficiency. After verification by the Bootstrap internal verification, we found that the calibration curves showed good agreement between the nomogram predicted and observed values. And the DCA results showed that the nomogram had a high clinical application. In conclusion, we constructed and validated the nomogram to predict CI-AKI risk after PCI in patients with ACS and DM. The model can provide a scientific reference for predicting the occurrence of CI-AKI and improving the prognosis of patients.

## 1. Introduction

Acute coronary syndrome (ACS) is an acute ischemic heart syndrome caused by the rupture of the atherosclerotic plaque and subsequent thrombosis and is one of the leading causes of death worldwide [1, 2]. Percutaneous intervention (PCI) is currently the primary treatment for ACS patients, but 15% to 35% of patients develop contrast-induced acute kidney injury (CI-AKI) after PCI [3]. CI-AKI is characterized by a sharp decline in renal function and is a risk factor for increased mortality after PCI [4]. CI-AKI is the third leading cause of hospital-acquired kidney damage after surgery and nephrotoxic drug, seriously affecting patients' quality of life and prognosis [5]. With the development of medical technology, although people's understanding of CI-AKI has been further improved, there is still no effective treatment method. Therefore, early identification of the high risk of CI-AKI and timely intervention are of great significance for the prognosis of ACS patients after PCI.

Studies have shown that advanced age, hypertension, diabetes mellitus (DM), hyperuricemia, and the basic renal function are the influencing factors of CI-AKI in ACS patients [6–8]. In China, 37.6% of patients with ACS have DM [9]. Insulin resistance (IR) is a primary pathophysiological defect in patients with type 2 diabetes and an important risk factor for DM and cardiovascular disease [10, 11]. The homeostasis model assessment of insulin resistance (HOMA-IR) is currently commonly used to assess IR [12]. Although the hyperinsulinemic-euglycemic clamp is the gold standard for measuring IR, it is not commonly used in clinical practice due to its complexity and being time-consuming [13]. Triglyceride-glucose index (TyG index), calculated by fasting blood glucose and triglyceride, has attracted widespread attention as a novel evaluation index. Studies have shown that the TyG index strongly correlates with HOMA-IR and hyperinsulinemic-euglycemic clamp [14, 15]. A recent study by Park et al. showed that the TyG index was superior to HOMA-IR in predicting type 2 diabetes [16]. TyG index is not only significantly correlated with the long-term prognosis of patients with ACS but also can predict the adverse cardiovascular outcomes of patients with ACS complicated with DM after PCI [17, 18].

As a simple and accurate visualization tool, the nomogram has been widely used to predict the incidence of each patient endpoint event. Bo et al. developed a simple and practical nomogram for predicting the prognosis of ACS patients, which has potential clinical value [19]. However, there is still no study using nomogram for predicting CI-AKI after PCI in ACS patients with DM. Therefore, our study is aimed at developing and verifying a nomogram to predict the possibility of CI-AKI after PCI in patients with ACS complicated with DM and to provide scientific reference for clinical treatment decisions and the prevention of CI-AKI.

## 2. Methods

### 2.1. Study Population and Design

This study is based on the atherosclerotic cardiovascular disease (ASCVD) database of the Affiliated Hospital of Xuzhou Medical University. This study selected patients diagnosed with ASCVD combined with DM from August 2018 to December 2021. The Medical Research Ethics Committee approved this study of the Affiliated Hospital of Xuzhou Medical University (approval number XYFY2022-KL093-01). Because the study was a single-center retrospective study, the review committee abandoned the requested written informed consent.

Inclusion criteria were as follows: (i) patients diagnosed with ACS according to 2020 ESC guidelines for the management of ACS [20], which included ST-segment elevation myocardial infarction (STEMI), non-ST-segment elevation myocardial infarction (NSTEMI), and unstable angina (UA); (ii) patients with a previous history of DM; and (iii) PCI was performed during this hospitalization.

Exclusion criteria were as follows: (i) patients with incomplete clinical data, (ii) severe heart valve disease requiring surgical treatment, (iii) patients with acute infection, (iv) a history of malignant tumor, (v) severe liver insufficiency, and (vi) severe kidney disease (eGFR < 15 mL/min,·1.73 m^2^).

**Figure 1:**
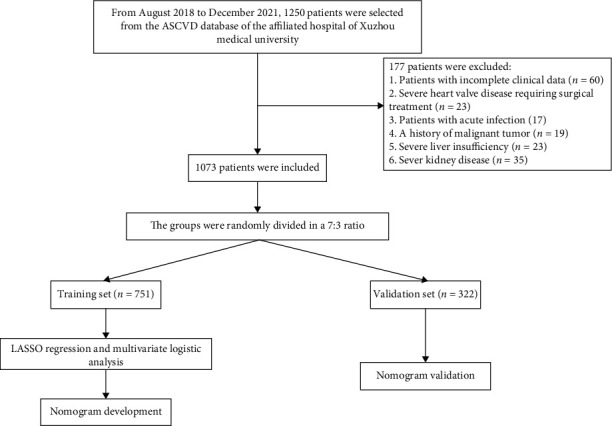
Flow chart of the inclusion and exclusion process of patients with ASCVD and diabetes mellitus. Abbreviation: ASCVD: atherosclerotic cardiovascular disease.

From August 2018 to December 2021, 1250 patients were selected from the ASCVD database, and the total number of patients included was 1073 according to the exclusion criteria. The specific inclusion and exclusion process is shown in Figure 1.

### 2.2. Clinical Endpoints

This study adopted the CI-AKI diagnostic criteria proposed in “Kidney Disease: Improving Global Outcomes (KDIGO)” in 2018: serum creatinine (Scr) levels increased by ≥26.5 mmol/L (0.3 mg/dL) or at least 50% from baseline within one week after the use of contrast agent [21].

### 2.3. Predictor Variables

By reviewing the literature, we collected some influential factors that may affect the occurrence of CI-AKI after PCI in patients with ACS combined with DM [22–24]. Clinical data for all patients were collected from the ASCVD database, including 54 variables such as patient's demographic data (age, gender, body mass index (BMI), systolic blood pressure (SBP), and diastolic blood pressure (DBP)), past medical history (hypertension, coronary heart disease, myocardial infarction, chronic kidney disease (CKD), hyperuricemia, etc.), laboratory indicators (fasting blood glucose (FBG), fasting total triglycerides (TG), low-density lipoprotein cholesterol (LDL-C), left ventricular ejection fraction (LVEF), left ventricular end-diastolic diameter (LVEDD), etc.), medication use during hospitalization (aspirin, statins, angiotensin-converting enzyme inhibitor (ACEI), insulin, anticoagulation, etc.), and PCI data (contrast agents, number of stents, coronary artery stenosis, etc.). All hematological parameters were completed 24 hours after admission, and renal function was rechecked within 7 days after PCI. The TyG index is calculated by fasting TG level (mg/dL) × FBG level (mg/dL)/2.

### 2.4. Statistical Analysis

This study used SPSS 22.0 and R version 3.6.4 for statistical analysis. Categorical variables were expressed as frequencies and percentages (%), and comparisons between groups were made by the Chi-squared test. Continuous variables were assessed by the Shapiro-Wilk test and Levene's test to assess normality and homogeneity of variance, respectively. If the date met normal distribution, it was expressed as mean ± standard deviation (*x* ± *s*), and comparisons between groups were made by the two independent samples *T*-test. If not, median (*M*) and interquartile ranges *M*(P25, P75) were used, and comparisons between groups were made with a nonparametric test. *P* < 0.05 indicated statistical differences. The study indexes were included in the least absolute shrinkage and selection operator (LASSO) regression for screening nonzero coefficient characteristics and the multivariate logistic regression analysis to screen out the independent predictors of CI-AKI. The above factors were introduced into R software, and the nomogram was drawn. We use the Bootstrap method for internal validation. The area under the receiver operating characteristic curve (AUC-ROC) and the calibration curve were used to assess the discriminatory ability and calibration of the model, respectively. Finally, the clinical value of the model was assessed by the decision curve analysis (DCA) curves.

## 3. Results

### 3.1. Baseline Characteristics

A total of 1073 patients with ASCVD combined with DM were included in this study. Patients were divided into the non-CI-AKI and CI-AKI groups based on the presence or absence of endpoint events. Comparing the baseline characteristics of the two groups, we found that the group with CI-AKI had a higher proportion of ST-segment elevation myocardial infarction (STEMI) and non-ST-segment elevation myocardial infarction (NSTEMI), was older, had a greater proportion of recent ACS and multivessel coronary artery disease, and was more likely to have a combination of hyperuricemia and hyperlipidemia. In addition, the CI-AKI group also had higher low-density lipoprotein cholesterol (LDL-C), TG, FBG, TyG index, and lower eGFR levels. For medications, evolocumab was used at a lower rate in the CI-AKI group. All the above differences were statistically significant, as shown in Table 1.

### 3.2. LASSO Regression and Multivariate Logistic Analyses

**Figure 2:**
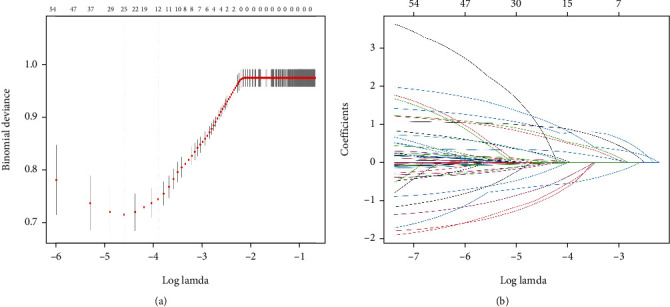
LASSO regression model screening predictors of CI-AKI. (a) LASSO regression model cross-validation plot. Draw a vertical line at the optimum with the minimum criterion and 1se of the minimum criterion. When *λ* = 0.0201, we get 12 variables for further analysis. (b) Coefficient profile plot of predictors. Finally, nine variables were selected at the optimal lambda, which is consistent with the results selected by logistic regression. Abbreviation: CI-AKI: contrast-induced acute kidney injury.

LASSO regression results showed the subtypes of ACS, age, multivessel coronary artery disease, hyperuricemia, hyperlipidemia, LDL-C, FBG, TyG index, eGFR, and evolocumab are important predictors of CI-AKI after PCI in patients with ACS and DM (see Figure 2). As shown in Table 2, including these indicators, in a multivariate logistic analysis, it showed that the subtypes of ACS, age, multivessel coronary artery disease, hyperuricemia, LDL-C, FBG, TyG index, and eGFR were independent risk factors for the occurrence of CI-AKI after PCI in patients with ACS combined with DM.

### 3.3. Clinical Features of the Training Set and Validation Set

To prevent overfitting of the nomogram in the analysis, we randomly divided ASCVD patients into the training and validation sets in the ratio of 7 : 3. Except for coronary artery disease, there was no difference between the training and validation sets for any other baseline characteristics. This illustrates that the division of our dataset is reasonable and comparable, as shown in Table 3.

### 3.4. Development of the Nomogram

**Figure 3:**
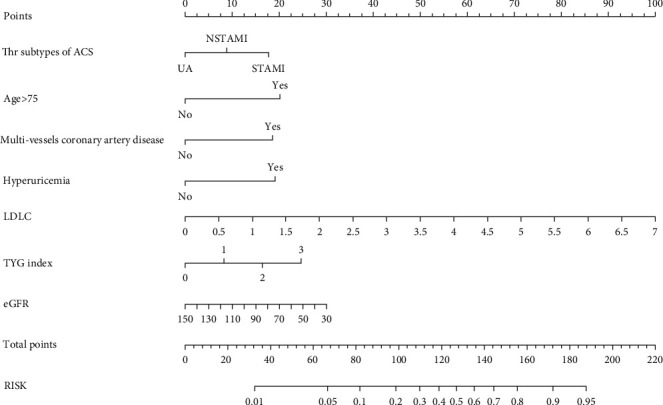
Nomogram used for predicting CI-AKI in patients with ASCVD and diabetes mellitus. Abbreviations: ACS: acute coronary syndrome; UA: unstable angina; STAMI: ST-segment elevation myocardial infarction; NSTAMI: non-ST-segment elevation myocardial infarction; LDL-C: low-density lipoprotein cholesterol; TyG: triglyceride-glucose; eGFR: estimated glomerular filtration rate.

**Figure 4:**
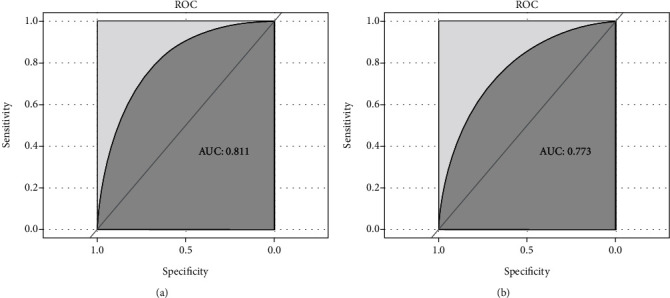
ROC curves of clinical prediction models were drawn based on the data of the training set (a) and validation set (b). Abbreviation: AUC: the area under the receiver operating characteristic.

**Figure 5:**
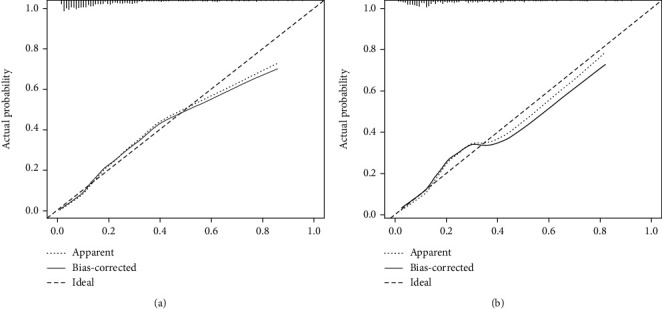
Calibration curve of the nomogram on the data of the training set (a) and validation set (b).

**Figure 6:**
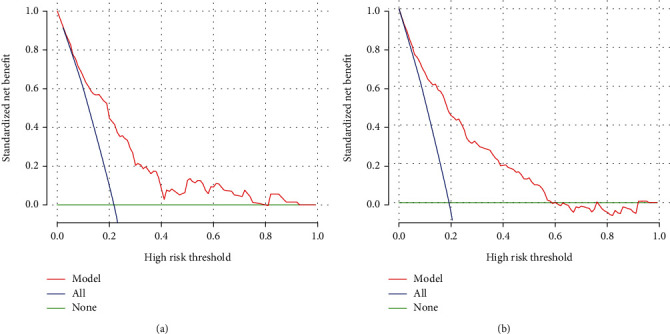
Evaluation of clinical validity of predictive models on the data of the training set (a) and validation set (b).

Nomogram was plotted according to the relative weights of each risk factor in the multivariate logistic regression analysis (see Figure 3). Regarding the validation of the nomogram, we proceed through the following three steps. First, we validate the model's discriminative ability by plotting the ROC curve (Figure 4). The AUC of the training and validation sets are 0.811 (95% CI: 0.766-0.844) and 0.773 (95% CI: 0.712-0.829), respectively. This indicates that the model has an excellent discriminative ability. Second, we used the Bootstrap self-sampling method with *B* = 1000 repetitions and plotted the calibration curves for the training and validation sets (see Figure 5). The results show that the predicted probability of the model output is in good agreement with the true occurrence probability, and the model calibration is good. Finally, to verify the clinical validity of the model, we plotted DCA curves (see Figure 6). The results show that the net benefit of the nomogram is significantly higher in the training and validation sets than in the two extreme cases. Therefore, the nomogram has good clinical significance.

## 4. Discussion

Studies have shown that the risk of CI-AKI after PCI in ACS patients was 16.4%-21.2%, which is consistent with the 19.85% in our study [25, 26]. In this single-center retrospective study, we found that the subtypes of ACS, age, multivessel coronary artery disease, hyperuricemia, LDL-C, TyG index, and eGFR were independent risk factors for the occurrence of CI-AKI after PCI in patients with ACS combined with DM, and we created the acceptable accuracy nomogram model with internal validation testing.

Regarding the demographic data, we found that the subtypes of ACS, age, and hyperuricemia were independent risk factors for developing CI-AKI after PCI in patients with ACS combined with DM. Tsai et al. studied the development of AKI after PCI in 985,737 patients and found that STEMI was a favorable predictor [27]. The specific pathological mechanism of AKI after PCI in STEMI patients has not been fully elucidated. We speculate that it is related to the lack of renal perfusion, inflammation, and endothelial injury [28, 29]. With the increase of age, the renal function of elderly patients also gradually declines, and the glomerular filtration rate decline of patients over 40 years old can reach 1 mL/min per year [30]. AKI also increases the incidence of renal and cardiovascular adverse events, with a 90-day mortality rate of 26.2% [31]. The study by Vaara et al. showed that age is an independent predictor of AKI, which is consistent with our finding [31]. Hyperuricemia can increase oxidative stress, proliferate vascular smooth muscle cells, and increase the release of proinflammatory substances, resulting in renal impairment [32]. A study shows that hyperuricemia is an independent predictor of CI-AKI and mortality in patients after PCI [33]. This study found that patients with multivessel coronary artery disease had a higher risk of developing AKI, which we speculate is related to the need for readmission and repeated procedures in these patients.

In this study, LDL-C in the CI-AKI group were higher than those in the non-AKI group, which was an independent predictor for the occurrence of CI-AKI. LDL-C is involved in endothelial dysfunction, injury, vasoconstriction, and inflammation, which are closely related to the occurrence of CI-AKI [34]. Liu et al. studied 3236 patients undergoing PCI and found that LDL-C level was an independent risk factor for CI-AKI, which was consistent with our findings [35]. A study has found that the use of proprotein convertase subtilisin/kexin type 9 (PCSK9) inhibitors and statins is promising to prevent the occurrence of CI-AKI [36]. In the multivariate analysis of this paper, hyperlipidemia and TG were not statistically significant, which may be related to the drug use of evolocumab and statins. We hope that this finding can be further verified in future studies. And eGFR is an important indicator of renal function. Studies have shown that eGFR can help identify the risk group for AKI and is an independent risk factor for CI-AKI occurrence in elderly patients after PCI [5, 37]. Insulin resistance (IR) is closely associated with kidney damage, and the TyG index is expected to be an alternative indicator of IR as an easily accessible and accurate indicator [38, 39]. Regarding the mechanism of insulin resistance damage to the kidney, it is considered to be related to the following reasons [40, 41]: (1) IR and oxidative stress are closely related, and the increase in the production of free radicals causes damage to the kidney. (2) IR can increase insulin in the body, which can be combined with abnormal glucose metabolism and lipid metabolism at the same time, and eventually metabolic diseases such as obesity, DM, and cardiovascular disease can aggravate kidney damage. This study showed that a total of 88 (41.3%) individuals developed CI-AKI when the TyG index was ≥9.4, and the TyG index was an important predictor of the occurrence of CI-AKI. Qin et al. found that a high TyG index was highly correlated with the increased occurrence of CI-AKI and was an important risk factor for CI-AKI, which supports our conclusion [26].

### 4.1. Study Limitations

Our article has some limitations as follows: (1) This is a single-center retrospective study with a lack of external validation. It is hoped that a multicenter, prospective study will be conducted in the future to confirm this finding further. (2) This article did not follow up with patients after discharge, including drug use and all-cause mortality. In the future, it is hoped that the overall performance of the prediction model can be further improved by establishing a complete follow-up system.

## 5. Conclusion

We constructed and validated the nomogram to predict the risk of CI-AKI after PCI in patients with ACS and DM. The model can provide a scientific reference for predicting the occurrence of CI-AKI and improving the prognosis of patients. To ensure generality, this model requires external validation.

## Figures and Tables

**Table 1:** Baseline characteristics of the non-CI-AKI group and CI-AKI group.

Variables	Non-CI-AKI group (*n* = 860)	CI-AKI group (*n* = 213)	*P* value
The subtypes of ACS (*n*, %)			<0.001
UA	491 (57.1%)	70 (32.9%)	
STAMI	185 (21.5%)	78 (36.6%)	
NSTAMI	184 (21.4%)	65 (30.5%)	
Age	65 (56,71)	66 (57,75.5)	0.048
Age > 75 (*n*, %)	125 (14.5%)	82 (38.5%)	<0.001
Gender (*n*, %)			0.203
Male	537 (62.4%)	143 (67.1%)	
Female	323 (37.6%)	70 (32.9%)	
BMI (kg/m^2^)	24.5 (23-26.775)	24.3 (22.75-26.20)	0.178
SBP (mmHg)	134 (124-145)	132 (120-146)	0.264
DBP (mmHg)	79 (72-86)	80 (72-87)	0.323
Smoking (*n*, %)			0.086
No	677 (78.7%)	156 (73.2%)	
Yes	183 (21.3%)	57 (26.8%)	
Drinking (*n*, %)			0.18
No	738 (85.8%)	175 (82.2%)	
Yes	122 (14.2%)	38 (17.8%)	
Past medical history (*n*, %)			
Coronary heart disease	837 (97.3%)	203 (95.3%)	0.126
PCI surgery history	505 (58.7%)	130 (61%)	0.539
The recent ACS	192 (22.3%)	46 (21.6.0%)	0.819
History of cerebral infarction	263 (30.6%)	79 (37.1%)	0.068
History of unstable angina	499 (58.0%)	110 (51.6%)	0.092
History of stable angina	7 (0.8%)	0 (0.0%)	0.186
Multivessel coronary artery disease	536 (62.3%)	173 (81.2%)	<0.001
Hyperuricemia	242 (28.1%)	94 (44.1%)	<0.001
Ischemic stroke	178 (20.7%)	40 (18.8%)	0.533
Peripheral artery stenosis	23 (2.7%)	2 (0.9%)	0.133
Hypertension	474 (55.1%)	131 (61.5%)	0.092
Hyperlipidemia	48 (5.6%)	20 (9.4%)	0.041
Chronic kidney disease	69 (8.0%)	11 (5.2%)	0.155
Family history of CKD	23 (2.7%)	4 (1.9%)	0.506
Culprit vessel (*n*, %)			
Left anterior descending	582 (67.7%)	154 (72.3%)	0.193
Left circumflex	341 (39.7%)	84 (39.4%)	0.954
Right coronary artery	394 (45.8%)	98 (46%)	0.959
Left main	3 (0.3%)	2 (0.9%)	0.258
Stent number per patient (*n*, %)			0.165
1	691 (80.3%)	162 (76.1%)	
≥2	169 (19.7%)	51 (23.9%)	
Contrast medium > 100 (mL)	454 (52.8%)	128 (60.1%)	0.055
Hematological index			
LDL-C (mmol/L)	2.015 (1.58-2.6175)	2.7 (2.045-3.39)	0.001
HDL-C (mmol/L)	0.97 (0.81-1.14)	0.93 (0.825-1.145)	0.35
TC (mmol/L)	3.77 (3.1625-4.5075)	3.93 (3.215-4.845)	0.114
TG (mmol/L)	1.4 (1.06-1.9675)	1.61 (1.265-2.285)	0.002
FBG (mmol/L)	7.165 (5.98-8.87)	7.32 (6.455-9.355)	0.006
TyG index			<0.001
≤8.55	163 (19%)	19 (8.9%)	
8.556-8.98	243 (28.3%)	53 (24.9%)	
8.99-9.39	223 (25.9%)	53 (24.9%)	
≥9.40	231 (26.9%)	88 (41.3%)	
ALT (U/L)	25 (19-37)	29 (16.5-48.5)	0.213
AST (U/L)	32 (18-120)	40 (22-112.5)	0.051
Creatine kinase (U/L)	120 (66-201)	148 (72.5-769.5)	0.061
Troponin (ng/L)	13.4 (3-135.3075)	25.1 (5.62-546)	0.05
HbA1c (%)	7.3 (6.6-8.6)	7.26 (6.61-8.41)	0.656
Scr (umol/L)	62 (53-75)	62 (53-74)	0.693
eGFR (mL/min)	98.365 (90.0025-106.8325)	88.19 (78.81-97.605)	<0.001
Medications used before surgery			
Statin (*n*, %)	850 (98.8%)	210 (98.6%)	0.769
Ezetimibe (*n*, %)	159 (18.5%)	35 (16.4%)	0.485
Apolizumab (*n*, %)	162 (18.8%)	27 (12.7%)	0.035
Aspirin (*n*, %)	855 (99.4%)	211 (99.1%)	0.562
Clopidogrel (*n*, %)	293 (34.1%)	82 (38.5%)	0.225
Ticagrelor (*n*, %)	526 (61.2%)	129 (60.6%)	0.872
ACEI/ARB (*n*, %)	254 (29.5%)	71 (33.3%)	0.28
ARNI (*n*, %)	162 (18.8%)	52 (24.4%)	0.068
Beta-blockers (*n*, %)	584 (67.9%)	136 (63.8%)	0.259
Oral hypoglycemic drugs	500 (58.1%)	110 (51.6%)	0.087
Insulin (*n*, %)	104 (12.1%)	16 (7.5%)	0.058
Anticoagulant (*n*, %)	101 (11.7%)	21 (9.9%)	0.438

Abbreviations: UA: unstable angina; STAMI: ST-segment elevation myocardial infarction; NSTAMI: non-ST-segment elevation myocardial infarction; BMI: body mass index; SBP: systolic blood pressure; DBP: diastolic blood pressure; PCI: percutaneous coronary intervention; ACS: acute coronary syndrome; CKD: chronic kidney disease, LDL-C: low-density lipoprotein cholesterol; HDL-C: high-density lipoprotein cholesterol; TC: total cholesterol; TG: triglyceride; FBG: fast blood glucose; TyG: triglyceride-glucose; ALT: alanine transaminase; AST: aspartate aminotransferase; HbA1c: glycated hemoglobin A; Scr: serum creatinine; eGFR: estimated glomerular filtration rate; ACEI: angiotensin-converting enzyme inhibitor; ARB: angiotensin receptor blocker; ARNI: angiotensin receptor neprilysin inhibitor.

**Table 2:** Multivariate logistic analysis for the CI-AKI after PCI.

Variables	*β*	*sχ*	Wald *χ*^2^	Multivariate analysis	*P* value
				OR (95% CI)	
The subtypes of ACS					<0.001
UA					
STAMI	0.843	0.211	16.021	2.365 (1.570,3.563)	
NSTAMI	0.761	0.214	12.608	2.177 (1.431, 3.312)	
Age > 75, years	0.888	0.211	17.72	2.451 (1.624, 3.700)	<0.001
The recent ACS	-0.387	0.249	2.425		0.119
Multivessel coronary artery disease	0.879	0.209	17.634	2.377 (1.581, 3.574)	<0.001
Hyperuricemia	1.039	0.216	23.207	2.363 (1.653, 3.377)	<0.001
Hyperlipidemia	0.192	0.351	0.3	1.127 (0.573, 2.215)	0.584
LDL (mmol/L)	0.626	0.101	38.302	1.911 (1.570, 2.327)	<0.001
TG (mmol/L)	-0.123	0.112	1.21		0.271
FBG (mmol/L)	-0.061	0.04	2.36	0.953 (0.886, 1.025)	0.124
TyG index					0.002
≤8.55			14.967		
8.556-8.98	0.777	0.322	5.81	2.027 (1.087, 3.778)	
8.99-9.39	0.853	0.345	6.127	2.033 (1.072, 3.854)	
≥9.40	1.598	0.419	14.545	3.770 (1.936, 7.339)	
eGFR (mL/min)	-0.017	0.005	11.366	0.983 (0.973, 0.992)	0.001
Medications					
Apolizumab	-0.373	0.252	2.194	0.681 (0.417, 1.113)	0.139

Abbreviations: UA: unstable angina; STAMI: ST-segment elevation myocardial infarction; NSTAMI: non-ST-segment elevation myocardial infarction; ACS: acute coronary syndrome; LDL-C: low-density lipoprotein cholesterol; TG: triglyceride; FBG: fast blood glucose; TyG: triglyceride-glucose; eGFR: estimated glomerular filtration rate.

**Table 3:** Baseline characteristics of training and validation sets.

Variables	Training (*n* = 751)	Validation set (*n* = 322)	*P*
The subtypes of ACS (*n*, %)			0.085
UA	380 (51.0%)	181 (55.2%)	
STAMI	197 (26.4%)	66 (20.1%)	
NSTAMI	168 (22.6%)	81 (24.7%)	
Age	65 (57,73)	64 (56,71)	0.187
Age > 75 (*n*, %)	153 (20.5%)	54 (16.5%)	0.119
Gender (*n*, %)			0.905
Male	473 (63.5%)	207 (63.1%)	
Female	272 (36.5%)	121 (36.9%)	
BMI (kg/m^2^)	24.5 (22.9-26.7)	24.5 (23.025-27.075)	0.470
SBP (mmHg)	133 (123-146)	135 (122-145)	0.711
DBP (mmHg)	80 (72-86)	79 (71.25-86)	0.419
Smoking (*n*, %)			0.593
No	575 (77.2%)	258 (78.7%)	
Yes	170 (22.8%)	70 (21.3%)	
Drinking (*n*, %)			0.839
No	635 (85.2%)	278 (84.8%)	
Yes	110 (14.8%)	50 (15.2%)	
Past medical history (*n*, %)			
Coronary heart disease	730 (98.0%)	310 (94.5%)	0.002
PCI surgery history	455 (61.1%)	180 (54.9%)	0.057
The recent ACS	165 (22.1%)	73 (22.3%)	0.969
History of cerebral infarction	241 (32.3%)	101 (30.8%)	0.614
History of unstable angina	427 (57.3%)	182 (55.5%)	0.578
History of stable angina	5 (0.7%)	2 (0.6%)	0.908
Multivessel coronary artery disease	489 (65.6%)	220 (67.1%)	0.647
Hyperuricemia	231 (31.0%)	105 (32.0%)	0.744
Ischemic stroke	154 (20.7%)	64 (19.5%)	0.664
Peripheral artery stenosis	21 (2.8%)	4 (1.2%)	0.11
Hypertension	420 (56.4%)	185 (56.4%)	0.994
Hyperlipidemia	50 (6.7%)	18 (5.5%)	0.449
Chronic kidney disease	61 (8.2%)	19 (5.8%)	0.169
Family history of CKD	20 (2.7%)	7 (2.1%)	0.596
Culprit vessel (*n*, %)			
Left anterior descending	507 (68.1%)	229 (69.8%)	0.566
Left circumflex	287 (38.5%)	138 (42.1%)	0.273
Right coronary artery	350 (47.0%)	142 (43.3%)	0.264
Left main	3 (0.4%)	2 (0.6%)	0.646
Stent number per patient (*n*, %)			0.11
1	602 (80.8%)	251 (76.5)	
≥2	143 (19.2%)	77 (23.5%)	
Contrast medium > 100 (mL)	414 (55.6%)	168 (51.2%)	0.188
Hematological index			
LDL-C (mmol/L)	2.11 (1.605-2.81)	2.1 (1.6425-2.8175)	0.641
HDL-C (mmol/L)	0.97 (0.81-1.14)	0.96 (0.83-1.14)	0.765
TC (mmol/L)	3.79 (3.145-4.545)	3.835 (3.24-4.5375)	0.422
TG (mmol/L)	1.42 (1.07-2)	1.495 (1.11-2.0475)	0.415
FBG (mmol/L)	7.25 (6.115-9.035)	7.14 (6.065-8.8225)	0.748
TyG index			0.71
≤8.55	131 (17.6%)	51 (15.5%)	
8.556-8.98	203 (27.2%)	93 (28.4%)	
8.99-9.39	186 (25%)	90 (27.4%)	
≥9.40	225 (30.2%)	94 (28.7%)	
ALT (U/L)	25 (18-39)	25.5 (18-39)	0.629
AST (U/L)	36 (19-117)	30.5 (19-122.75)	0.557
Creatine kinase (U/L)	126 (66-230.5)	119.5 (70-209.25)	0.908
Troponin (ng/L)	14 (3.365-274.5)	14 (3.0475-130.25)	0.592
HbA1c (%)	7.31 (6.61-8.61)	7.21 (6.61-8.4575)	0.596
Scr (umol/L)	62 (53-75)	62 (54-74)	0.565
eGFR (mL/min)	97.1 (86.085-106.08)	96.505 (87.27-105.105)	0.604
Medications used before surgery			
Statin (*n*, %)	735 (98.7%)	325 (99.1%)	0.555
Ezetimibe (*n*, %)	136 (18.3%)	58 (17.7%)	0.822
Apolizumab (*n*, %)	131 (17.6%)	58 (17.7%)	0.969
Aspirin (*n*, %)	739 (99.2%)	327 (99.7%)	0.348
Clopidogrel (*n*, %)	270 (36.2%)	105 (32.0%)	0.181
Ticagrelor (*n*, %)	442 (59.3%)	213 (64.9%)	0.083
ACEI/ARB (*n*, %)	225 (30.2%)	100 (30.5%)	0.925
ARNI (*n*, %)	151 (20.3%)	63 (19.2%)	0.689
Beta-blockers (*n*, %)	504 (67.7%)	216 (65.9%)	0.564
Oral hypoglycemic drugs	424 (56.9%)	186 (56.7%)	0.95
Insulin (*n*, %)	83 (11.1%)	37 (11.3%)	0.947
Anticoagulant (*n*, %)	83 (11.1%)	39 (11.9%)	0.722

Abbreviations: UA: unstable angina; STAMI: ST-segment elevation myocardial infarction; NSTAMI: non-ST-segment elevation myocardial infarction; BMI: body mass index; SBP: systolic blood pressure; DBP: diastolic blood pressure; PCI: percutaneous coronary intervention; ACS: acute coronary syndrome; CKD: chronic kidney disease, LDL-C: low-density lipoprotein cholesterol; HDL-C: high-density lipoprotein cholesterol; TC: total cholesterol; TG: triglyceride; FBG: fast blood glucose; TyG: triglyceride-glucose; ALT: alanine transaminase; AST: aspartate aminotransferase; HbA1c: glycated hemoglobin A; Scr: serum creatinine; eGFR: estimated glomerular filtration rate; ACEI: angiotensin-converting enzyme inhibitor; ARB: angiotensin receptor blocker; ARNI: angiotensin receptor neprilysin inhibitor.

## Data Availability

The raw data supporting the conclusions of this article will be made available by the authors without undue reservation.
